# Increased Production of Interleukin-10 and Tumor Necrosis Factor-Alpha in Stimulated Peripheral Blood Mononuclear Cells after Inhibition of S100A12

**DOI:** 10.3390/cimb44040117

**Published:** 2022-04-12

**Authors:** Huang-Pin Wu, Chien-Ming Chu, Pi-Hua Liu, Shaw-Woei Leu, Shih-Wei Lin, Han-Chung Hu, Kuo-Chin Kao, Li-Fu Li, Chung-Chieh Yu

**Affiliations:** 1 Division of Pulmonary, Critical Care and Sleep Medicine, Chang Gung Memorial Hospital, Keelung 20401, Taiwan; whanpyng@cgmh.org.tw (H.-P.W.); rocephen@cgmh.org.tw (C.-M.C.); lfp3434@cgmh.org.tw (L.-F.L.); 2 College of Medicine, Chang Gung University, Taoyuan 33302, Taiwan; swleu@cgmh.org.tw (S.-W.L.); ec108146@cgmh.org.tw (S.-W.L.); h3226@cgmh.org.tw (H.-C.H.); kck0502@cgmh.org.tw (K.-C.K.); 3 Clinical Informatics and Medical Statistics Research Center, College of Medicine, Chang Gung University, Taoyuan 33302, Taiwan; phliu@mail.cgu.edu.tw; 4 Division of Endocrinology and Metabolism, Department of Internal Medicine, Linkou Chang Gung Memorial Hospital, Taoyuan 33305, Taiwan; 5 Department of Pulmonary and Critical Care Medicine, Chang Gung Memorial Hospital, Taoyuan 33305, Taiwan

**Keywords:** S100A12, RAGE, sepsis, interleukin-10, tumor necrosis factor-α

## Abstract

Sepsis may induce immunosuppression and result in death. S100A12 can bind to the receptor for advanced glycation end-products (RAGE) and Toll-like receptor (TLR)4 following induction of various inflammatory responses. It is unclear whether S100A12 significantly influences the immune system, which may be associated with sepsis-related mortality. We measured plasma S100A12 levels and cytokine responses (mean ± standard error mean) of lipopolysaccharide (LPS)-stimulated peripheral blood mononuclear cells (PBMCs) after S100A12 inhibition in healthy controls and patients with sepsis on days one and seven. Day one plasma soluble RAGE (sRAGE) and S100A12 levels in patients with sepsis were significantly higher than those in controls (2481.3 ± 295.0 vs. 1273.0 ± 108.2 pg/mL, *p* < 0.001; 530.3 ± 18.2 vs. 310.1 ± 28.1 pg/mL, *p* < 0.001, respectively). Day seven plasma S100A12 levels in non-survivors were significantly higher than those in survivors (593.1 ± 12.7 vs. 499.3 ± 23.8 pg/mL, *p* = 0.002, respectively). In survivors, plasma sRAGE levels were significantly decreased after 6 days (2297.3 ± 320.3 vs. 1530.1 ± 219.1 pg/mL, *p* = 0.009, respectively), but not in non-survivors. Inhibiting S100A12 increased the production of tumor necrosis factor (TNF)-α and interleukin (IL)-10 in stimulated PBMCs for both controls and patients. Therefore, S100A12 plays an important role in sepsis pathogenesis. S100A12 may competitively bind to TLR4 and RAGE, resulting in decreased IL-10 and TNF-α production.

## 1. Introduction

Sepsis is an infection-induced syndrome that is characterized by physiological, pathological, and biochemical abnormalities. The definition of sepsis has been revised in the 3rd International Consensus Definitions for Sepsis and Septic Shock (Sepsis-3) as a life-threatening organ dysfunction caused by a dysregulated host response to infection [1]. In sepsis pathogenesis, immune system failure and sepsis-induced immunosuppression may result in death [2]. One of the causes of this is high interleukin (IL)-10 production in sepsis [3,4,5]. An improved understanding of the mechanisms underlying this immunosuppression and immune defects may lead to the development of novel therapeutic strategies for patients with sepsis.

Pro-inflammatory cytokines are produced by immune cells upon stimulation with lipopolysaccharide (LPS), which binds to Toll-like receptor (TLR)4 and activate nuclear factor-kappa B (NF-κB). Recently, the receptor for advanced glycation end-products (RAGE) was found to bind to LPS [6]. The multi-ligand RAGE is regarded as a prototypic receptor for damage-associated molecular patterns (DAMPs), including high-mobility group box 1 (HMGB1) and advanced glycation end-products (AGE) [7]. RAGE is expressed at high levels in the lungs and at low levels in cells of the innate immune system, such as neutrophils, T and B lymphocytes, monocytes, macrophages, dendritic cells, and endothelial cells [8]. Engagement of RAGE leads to the activation of the NF-κB and mitogen-activated protein kinase pathways. RAGE activation can induce sterile inflammatory diseases, including diabetic nephropathy, hepatic injury, and diabetic atherosclerosis [8,9,10,11].

RAGE recognizes different ligands through its three-dimensional structure, and its putative ligands include AGE, HMGB1, and S100 proteins [12]. The soluble isoforms of RAGE function as decoy receptors and antagonize membrane RAGE ligand-binding. Thus, soluble RAGE (sRAGE) binds AGE or S100 in circulation and prevents the binding of AGE or S100 to RAGE in immune cells. S100A12 is an intracellular calcium-binding protein belonging to the S100 family. Once cell damage, infection, or inflammation occurs, S100A12 is released from immune cells into the extracellular compartment and acts as a pro-inflammatory signal and an endogenous TLR4 ligand [12,13]. Serum S100A12 has been identified as a valuable marker for predicting autoimmune hepatitis [14]. Compared with healthy volunteers, patients and neonates with sepsis display increased circulating S100A12 levels [15,16,17]. Higher plasma levels of S100A12 at admission have been reported to signify a higher risk of death in patients with septic shock [18], and were associated with subsequent occurrence of nosocomial infections through up-regulation of monocytic and granulocytic myeloid-derived suppressor cells, which contribute to T-cell dysfunction in patients with sepsis [16].

We hypothesized that S100A12 might influence the immune system because: (1) it is a ligand of RAGE and TLR4; and (2) it is related to T-cell function. In this context, the aims of this study were: (1) to compare plasma levels of S100A12 and other associated mediators between survivors and non-survivors with sepsis; (2) to compare serial change of levels of S100A12 and other associated mediators in survivors and non-survivors with sepsis; (3) to evaluate the effect of inhibiting S100A12 on cytokine production in stimulated peripheral blood mononuclear cells (PBMCs); and (4) to create a possible mechanism that modulates cytokine production. Therefore, this prospective observational study was designed using repeated blood sampling to determine the role of S100A12 in patients with sepsis.

## 2. Materials and Methods

### 2.1. Participants and Definitions

Between August 2016 and July 2018, 44 patients admitted to a 20-bed intensive care unit (ICU) in a regional teaching referral hospital for sepsis were enrolled in this study. To validate the experimental findings, 27 healthy controls who visited our health evaluation center for examination were enrolled.

Sepsis was defined as a suspected or documented infection with an acute increase (≧2) in Sequential Organ Failure Assessment points. Septic shock was defined as sepsis with a blood lactate level >18 mg/dL and hypotension that was unresponsive to fluid resuscitation, requiring vasopressors to maintain a mean arterial pressure ≧65 mmHg during the first 3 d following ICU admission. Respiratory failure was defined as a ventilation dysfunction requiring invasive ventilator support. Acute renal failure was diagnosed based on a rapidly rising serum creatinine level ≥0.5 mg/dL over the baseline value [19]. Jaundice was defined as hyperbilirubinemia (total bilirubin >2 mg/dL) whereas thrombocytopenia was defined as a platelet count <150,000/μL. Disease severity was assessed based on the Acute Physiology and Chronic Health Evaluation (APACHE) II score [20].

Standard treatment, according to the guidelines, was provided to all patients including initial fluid resuscitation, antibiotics use, vasopressor to keep blood pressure, renal replacement therapy for acute renal failure, and low tidal ventilation in acute respiratory distress syndrome according to patients’ conditions [21]. The Institutional Review Board at Chang Gung Memorial Hospital approved this study (103-7093B, 104-8013C), and informed consent was obtained from close relatives of the patients. Patients who survived for longer than 28 d after ICU admission were defined as survivors. All comorbidities and medical histories were recorded.

### 2.2. Plasma and Peripheral Blood Mononuclear Cell (PBMC) Preparation

PBMCs were used to determine the role of RAGE because they are the important immune cells that defend against pathogens in sepsis. Whole blood (10 mL) was obtained from each patient at 08:30 AM within 48 h of admission to the ICU and was immediately mixed with heparin. Whole blood from the controls was obtained at 08:00–08:30 AM and mixed with heparin. The day of the first blood sampling was defined as Day 1. The second blood sample was obtained on Day 7. Second blood sampling was not performed in the controls. Plasma samples were obtained from 2 mL of whole blood, and stored at −80 °C until use. PBMCs were isolated through differential centrifugation over Ficoll-Plaque (Amersham Biosciences, Uppsala, Sweden) from residual 8 mL of whole blood within 2 h of collection.

### 2.3. Measurement of Plasma Cytokine Levels

The plasma levels of sRAGE and S100A12 were measured using human enzyme-linked immunosorbent assay (ELISA) kits (R&D Systems, Inc., Minneapolis, MN, USA), according to the manufacturer’s instructions. Plasma AGE levels were measured using human ELISA kits (Cell Biolabs, Inc., San Diego, CA, USA), according to the manufacturer’s instructions. Plasma HMGB1 levels were measured using human ELISA kits (MyBioSource, Inc., San Diego, CA, USA), according to the manufacturer’s instructions.

### 2.4. Cell Culture

Total PBMCs (5 × 10^5^) were plated in three wells of a flat-bottomed 24-well plate (Nunclon, Aarhus, Denmark) in 1 mL sterile Roswell Park Memorial Institute 1640 tissue culture medium containing 5% heat-inactivated bovine serum and 1 mM sodium pyruvate. Cells in the 1st well were not stimulated or treated. Cells in the 2nd well were stimulated with 1 µg/mL LPS (Sigma, Burlington, MO, USA). Cells in the 3rd well were stimulated with 1 µg/mL LPS (Sigma, Burlington, MO, USA) and treated with 250 pg/mL neutralizing anti-S100A12 human antibody (R&D Systems, Inc., Minneapolis, MN, USA). The plate was incubated at 37 °C in 5% CO2 for 24 h. The supernatants of the culture wells were sampled and stored at −80 °C until use.

### 2.5. Measurement of Supernatant Cytokine Levels

Supernatant levels of tumor necrosis factor (TNF)-α and IL-10 were measured using human ELISA kits (Becton Dickinson, San Diego, CA, USA), according to the manufacturer’s instructions. The supernatant levels of IL-12 were measured using human ELISA kits (R&D Systems, Inc., Minneapolis, MN, USA), according to the manufacturer’s instructions. These cytokines were measured because they are known to be increased in sepsis [3,22].

### 2.6. Statistical Analysis

Statistical analysis was performed using the Statistical Package for the Social Sciences (SPSS) software V17.0 for Windows (SPSS Inc., Chicago, IL, USA). Data are presented as the mean ± standard error mean or frequencies (number of cases) and percentages. Differences in continuous variables between the two groups were analyzed using the independent-samples *t*-test, whereas differences in categorical variables were analyzed using the chi-squared test or Fisher’s exact test. Differences in continuous variables in the same participants were analyzed using the paired-sample *t*-test. Correlations between two continuous variables were compared using Pearson’s correlation test. Statistical significance was set at *p* < 0.05.

## 3. Results

Of the 44 enrolled patients with sepsis, 30 survived for 28 d and 14 died. A total of nine non-survivors died within 7 d, and one survivor was discharged from the ICU within 7 d. The clinical characteristics of the patients are presented in Table 1. There were no significant differences in age, sex, history, or source of infection between survivors and non-survivors. Non-survivors had higher APACHE II scores and higher percentages of acute renal failure and septic shock. The percentages of new arrhythmia, gastrointestinal bleeding, thrombocytopenia, jaundice, and bacteremia were similar between the two groups.

The mean age in the patient group was approximately 15 years older than that in the control group. Concerning the effect of age on the plasma levels of AGE, sRAGE, HMGB1, and S100A12, further correlation statistical analysis was performed. There was no correlation between age and Day 1 plasma levels of AGE, sRAGE, HMGB1, and S100A12 in both the control and patient groups (data not shown). In addition, since mortality was associated with acute renal failure and shock, the plasma levels of AGE, sRAGE, HMGB1, and S100A12 were used to analyze the presence and absence of acute renal failure and shock. There were no differences in Day 1 plasma levels of AGE, sRAGE, HMGB1, and S100A12 between the presence and absence of acute renal failure and shock (data not shown).

### 3.1. Plasma Levels of AGE, sRAGE, HMGB1, and S100A12 in Survivors, Non-Survivors, and Controls

Day 1 plasma levels of AGE, sRAGE, HMGB1, and S100A12 in non-survivors were similar to those in survivors (Table 2). On Day 7, plasma S100A12 levels in non-survivors were higher than those in survivors. There were no differences in Day 7 plasma levels of AGE, sRAGE, and HMBG1 between survivors and non-survivors. Day 1 plasma levels of sRAGE and S100A12 in patients with sepsis were significantly higher than those in controls. Day 1 plasma levels of AGE and HMGB1 in patients with sepsis were similar to those in controls.

### 3.2. Plasma Levels of AGE, sRAGE, HMGB1, and S100A12 between Days 1 and 7

There were 29 survivors and five non-survivors who had data on both Days 1 and 7. Plasma sRAGE levels were significantly decreased in these 34 patients with sepsis after 6 d (data not shown). The plasma levels of AGE, HMGB1, and S100A12 did not change in patients with sepsis after 6 d.

**Figure 1:**
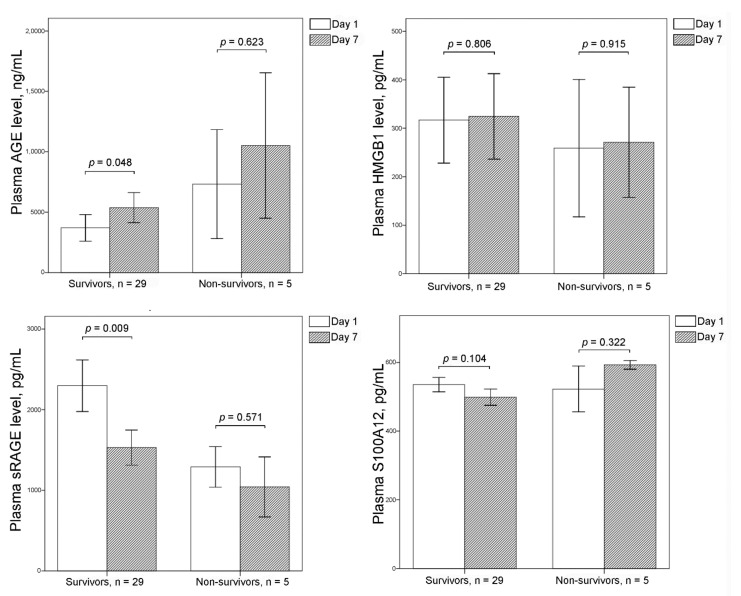
Plasma levels of advanced glycation endproducts (AGE), soluble RAGE (sRAGE), high-mobility group box 1 (HMGB1), and S100A12 on Days 1 and 7 are shown using bar charts with one standard error. In survivors, plasma AGE levels were significantly increased and plasma sRAGE levels were significantly decreased after 6 d. There were no changes in plasma levels of AGE and sRAGE in non-survivors. Plasma levels of HMGB1 and S100A12 did not change in survivors and non-survivors after 6 d.

In survivors, plasma AGE levels were significantly increased and plasma sRAGE levels were significantly decreased after 6 d (Figure 1). There were no changes in the plasma levels of AGE and sRAGE after 6 d in non-survivors. Plasma levels of HMGB1 and S100A12 did not change in survivors and non-survivors after 6 d.

### 3.3. Effect of Inhibiting S100A12 on Cytokine Production in Stimulated PBMCs

**Figure 2:**
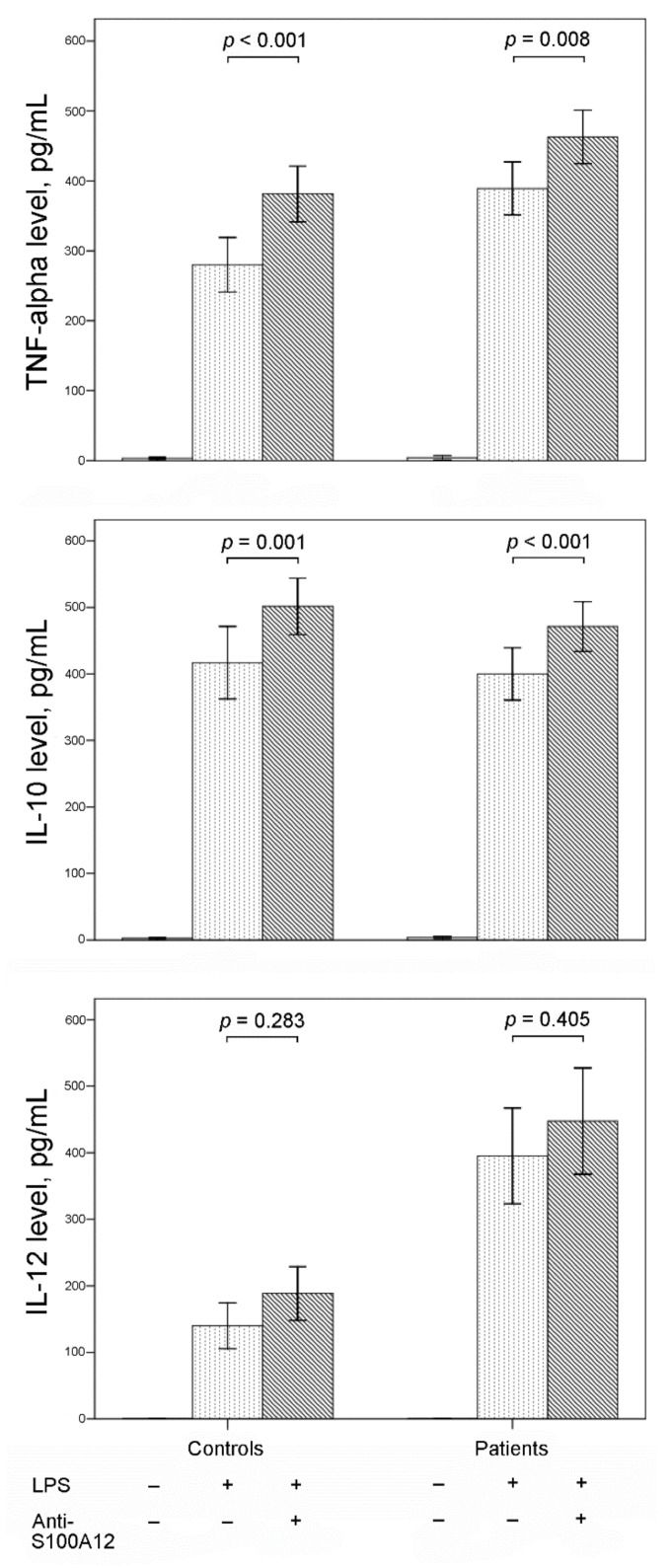
Bar charts with one standard error show supernatant levels of tumor necrosis factor (TNF)-α, interleukin (IL)-10, and IL-12 from peripheral blood mononuclear cells (PBMCs) with and without lipopolysaccharide (LPS) stimulation and anti-S100A12 treatment. Productions of TNF-α, IL-10, and IL-12 from PBMCs were significantly increased after LPS stimulation in both controls and patients with sepsis. Additional anti-S100A12 monoclonal antibodies increased the production of TNF-α and IL-10 in LPS-stimulated PBMCs from both controls and patients.

The production of TNF-α, IL-10, and IL-12 from PBMCs was significantly increased after LPS stimulation of PBMCs from both controls and patients with sepsis (Figure 2). Additional neutralizing with anti-S100A12 human antibody significantly increased the production of TNF-α and IL-10 in LPS-stimulated PBMCs from both controls and patients. There was no significant effect of additional neutralizing anti-S100A12 human antibody on IL-12 production in LPS-stimulated PBMCs from both controls and patients.

## 4. Discussion

This study found that patients with sepsis had higher plasma S100A12 levels than the controls. There was no difference in the plasma S100A12 levels between survivors and non-survivors on Day 1. Compared with survivors, non-survivors had higher plasma S100A12 levels on Day 7. This result may be due to a slight decrease in plasma S100A12 levels in survivors and a minor increase in plasma S100A12 levels in non-survivors after 6 d. Our results are the same as those reported by Achouiti et al. [15]. However, our study did not demonstrate that the non-survivors had significantly higher levels of S100A12 than survivors, as shown in the study by Dubois [18]. In critically ill patients admitted to the surgical ICU, serum S100A12 levels on admission were higher than those in controls [23]. As in our study, survivors and non-survivors had similar serum S100A12 levels on admission, but serum S100A12 levels in non-survivors were higher than those in survivors on Day 7 [23]. The opposite trend in serial plasma S100A12 levels between survivors and non-survivors may reflect persistently uncontrolled inflammation in non-survivors, resulting in death.

Compared with controls, patients with sepsis had higher plasma sRAGE levels. There was no difference in plasma sRAGE levels between survivors and non-survivors. This indicates that sRAGE could be a marker of sepsis but not a predictor of mortality in patients with sepsis. These results are similar to those of Matsumoto and Narvaez-Rivera [24,25]. Although Jabaudon et al. found that baseline plasma sRAGE levels were similar between sepsis patients and controls, baseline plasma sRAGE levels in patients with sepsis and acute respiratory distress syndrome were higher than those in controls [26]. In our study, septic patients with pneumonia who survived had significantly decreased plasma sRAGE levels after 6 d (Supplementary Table S1). This suggests that plasma sRAGE levels may be associated with lung injury and that plasma sRAGE levels might be used as a marker of pneumonia resolution.

**Figure 3:**
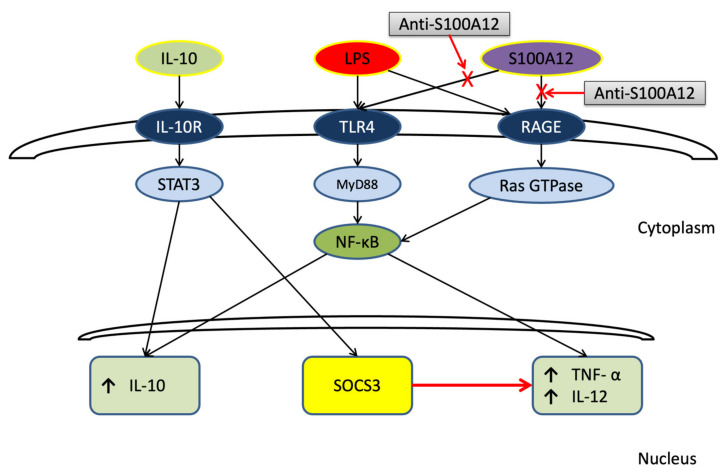
A proposed schematic mechanism that shows S100A12 may modulate tumor necrosis factor (TNF)-α and interleukin (IL)-10 production through lipopolysaccharide (LPS). LPS links Toll-like receptor (TLR) 4 to activate nuclear factor-kappa B (NF-κB) through myeloid differentiation primary response 88 (MYD88). The activated NF-κB in the cytoplasm is then translocated into the nucleus where it binds to specific sequences of DNA and increases IL-10, IL-12, and TNF-α gene expressions. Extracellular IL-10 binds to IL-10 receptors and activates signal transducer and activator of transcription (STAT)3. Then, STAT3 is translocated to the cell nucleus and induces IL-10 gene expression. Activating STAT3 signaling also induces suppressor of cytokine signaling (SOCS)3 to suppress IL-12 and TNF-α gene expression. Once RAGE is bound to LPS or S100A12, NF-κB is activated by the active form of rat sarcoma (Ras), Ras nucleotide guanosine triphosphate (GTP) hydrolases (GTPase). S100A12 may competitively bind to TLR4/RAGE and the affinity between S100A12 and TLR4/RAGE may be lower than that between LPS and TLR/4RAGE. This results in increased IL-10 and TNF-α production with S100A12 being inhibited.

The signaling pathways through which LPS links TLR4 to activate NF-κB through myeloid differentiation primary response 88 (MYD88) are known (Figure 3) [27,28,29,30]. Activated NF-κB in the cytoplasm is translocated into the nucleus, where it binds to specific sequences of DNA. NF-κB controls the expression of many genes, including IL-10, IL-12, and TNF-α. Extracellular IL-10 binds to IL-10 receptors and activates the signal transducer and activator of transcription (STAT)3. STAT3 is translocated to the cell nucleus and induces IL-10 gene expression but also regulates proliferation, apoptosis, invasion, metastasis, and angiogenesis in colorectal cancer (CRC) [31]. Recently, Polimeno et al. proposed alterations of the gut microbiota as a primer for tumorigenesis in patients with CRC, and demonstrated that STAT3 expression in different tissues was related to cancer severity [32]. Activating STAT3 signaling induces the suppressor of cytokine signaling (SOCS)3 to suppress IL-12 and TNF-α gene expression. However, the signaling cascades of RAGE, which ultimately activate NF-κB, remain largely unknown [33]. Once RAGE binds to ligands, NF-κB is activated bthe y active form of rat sarcoma (Ras), Ras nucleotide guanosine triphosphate hydrolases [34,35].

However, no study has determined the effect of S100A12 inhibition on cytokine responses in immune cells. Our study is the first to demonstrate that inhibiting S100A12 with neutralizing anti-S100A12 human antibody significantly increased TNF-α and IL-10 production in LPS-stimulated PBMCs from controls and patients with sepsis. However, Wen et al. found that S100A12 gene silencing decreased serum TNF-α levels in septic rats [36]. It is unclear whether decreased serum TNF-α can indicate that S100A12 inhibition results in decreased TNF-α production in LPS-stimulated PBMCs. Chung et al. had reported that S100A12 did not affect LPS-induced p38 phosphorylation or the NF-κB pathway in human PBMCs [37]. In a culture of normal human bronchial epithelial cells, LPS and S100A12 resulted in excessive secretion of sRAGE, TNF-α, IL-1β, and IL-6, and the cytokine production in LPS stimulation was higher than that in S100A12 [38]. In this study, the increase in IL-10 and TNF-α production after S100A12 inhibition may be due to receptor competition. LPS might have a higher affinity and response for binding to TLR4 and RAGE than S100A12. Other pathways influence cytokine production. Further studies are required to elucidate this aspect of immune signaling.

This study had two limitations. First, PBMCs were not stimulated by S100A12 alone. If an experiment with stimulation only by S100A12 was performed, the difference in cytokine production between the stimulatory effects of LPS and S100A12 could have been investigated. A direct impact of LPS and S100A12 on cytokine production can be proposed. Second, the proposed schema (Figure 3) was speculative because RAGE, TLR4, NF-κB, STAT3, and Ras were not measured. Well-controlled animal models or cell line studies are necessary to demonstrate our proposed hypothesis.

## 5. Conclusions

The S100A12 plays a key role in sepsis pathogenesis. It might be a candidate for diagnostic biomarkers to differentiate sepsis from no sepsis since the Day 1 and 7 plasma S100A12 levels in patients with sepsis were significantly higher than those in controls. Subsequently, the S100A12 levels were observed to be higher in the non-survivors than in the survivors, which suggests persistent high plasma S100A12 levels after 6 d predicted mortality. Through probable receptor competition, inhibition of S100A12 increased IL-10 production in LPS-stimulated PBMCs from both controls and patients with sepsis. This indicates that persistent expression of S100A12 by immune cells might not be a bad thing since S100A12 could prevent IL-10 overproduction known to be involved in the immunosuppression in the late stage of sepsis.

## Figures and Tables

**Table 1:** Clinical characteristics in survivors, non-survivors, and controls (number, mean ± standard error mean).

	Survivors (*n* = 30)	Non-Survivors (*n* = 14)	All Patients (*n* = 44)	Controls (*n* = 27)
Age (years old)	75.9 ± 1.8	74.7 ± 3.1	75.5 ± 1.6	60.3 ± 1.3 *
Male (%)	17 (56.7)	9 (64.3)	26 (50.1)	17 (63.0)
APACHE II score	20.8 ± 1.0	28.4 ± 2.0 ^†^	23.2 ± 1.1	
History (%)				
COPD	0 (0.0)	2 (14.3)	2 (4.5)	
Heart failure	5 (16.7)	0 (0.0)	5 (11.4)	
Hypertension	20 (66.7)	10 (71.4)	30 (68.2)	
Diabetes mellitus	12 (40.0)	7 (50.0)	19 (43.2)	
Old CVA	8 (26.7)	2 (14.3)	10 (22.7)	
ESRD	4 (13.3)	4 (28.6)	8 (18.2)	
Liver cirrhosis	3 (10.0)	2 (14.3)	5 (11.4)	
Active malignancy	2 (6.7)	0 (0.0)	2 (4.5)	
Infection source				
Pneumonia	21 (70.0)	8 (57.1)	29 (65.9)	
UTI	4 (13.3)	1 (7.1)	5 (11.4)	
Others	5 (16.7)	5 (35.8)	10 (22.7)	
Adverse event				
New arrhythmia	3 (10.0)	1 (7.1)	4 (9.1)	
GI bleeding	1 (3.3)	3 (21.4)	4 (9.1)	
Acute renal failure	8 (26.7)	10 (71.4) ^‡^	18 (40.9)	
Shock	13 (43.3)	12 (85.7) ^¶^	25 (56.8)	
Thrombocytopenia	8 (26.7)	7 (50.0)	15 (34.1)	
Jaundice	5 (16.7)	3 (21.4)	8 (18.2)	
Bacteremia	5 (16.7)	2 (14.3)	7 (15.9)	

Abbreviations: APACHE, Acute Physiology, and Chronic Health Evaluation; COPD, chronic obstructive pulmonary disease; CVA, cerebral vascular accident; ESRD, end-stage renal disease; UTI, urinary tract infection; GI, gastrointestinal. * *p* <0.001 compared with all patients with sepsis by independent-samples *t*-test; ^†^ *p* < 0.001 compared with survivors by independent-samples *t*-test; ^‡^ *p* = 0.008 compared with survivors by Fisher’s exact test; ^¶^ *p* = 0.010 compared with survivors by Fisher’s exact test.

**Table 2:** Plasma AGE, sRAGE, and HMGB1 levels (mean ± standard error mean) on Days 1 and 7 in survivors, non-survivors, and controls.

	Survivors	Non-Survivors	All Patients	Controls
Day 1	(*n* = 30)	(*n* = 14)	(*n* = 44)	(*n* = 27)
AGE, ng/mL	4335.9 ± 1249.4	3839.5 ± 1715.1	4177.9 ± 1001.0	2117.8 ± 833.4
sRAGE, pg/mL	2355.2 ± 314.9	2751.5 ± 650.0	2481.3 ± 295.0	1273.0 ± 108.2 *
HMGB1, pg/mL	306.3 ± 86.1	247.4 ± 56.8	287.6 ± 61.1	160.7 ± 54.0
S100A12, pg/mL	533.4 ± 20.2	523.6 ± 38.8	530.3 ± 18.2	310.1 ± 28.1 *
Day 7	(*n* = 29)	(*n* = 5)	(*n* = 34)	
AGE, ng/mL	5372.0 ± 1254.3	10517.0 ± 6020.9	6128.7 ± 1373.1	
sRAGE, pg/mL	1530.1 ± 219.1	1041.6 ± 371.9	1458.2 ± 195.2	
HMGB1, pg/mL	324.6 ± 88.1	271.0 ± 114.0	316.7 ± 76.6	
S100A12, pg/mL	499.3 ± 23.8	593.1 ± 12.7 ^†^	513.1 ± 21.1	

Abbreviations: AGE, advanced glycation end products; sRAGE, soluble receptor for AGE; HMGB1, high-mobility group box 1. * *p* < 0.001 compared with all patients with sepsis by independent-samples *t*-test; ^†^ *p* = 0.002 compared with survivors by independent-samples *t*-test.

## Data Availability

The datasets used and/or analyzed during the current study are available from the corresponding author upon reasonable request.

## Supplementary Materials

The following supporting information can be downloaded at: https://www.mdpi.com/article/10.3390/cimb44040117/s1, Table S1: Plasma AGE, sRAGE, and HMGB1 levels (mean ± standard error mean) on Days 1 and 7 in patients with complete data and sepsis due to pneumonia.
