# Supplementary material for: Increased Production of Interleukin-10 and Tumor Necrosis Factor-Alpha in Stimulated Peripheral Blood Mononuclear Cells after Inhibition of S100A12
Source: Curr Issues Mol Biol. 2022 Apr 12;44(4):1701–12. doi: 10.3390/cimb44040117 (PMC9164026; doi:10.3390/cimb44040117)
Supplement: Supplementary file 1 [file cimb-44-00117-s001.zip › cimb-1645071-supplementary.pdf]

Table S1. Plasma AGE, sRAGE, and HMGB1 levels (mean ± standard error mean) on Days 1 and 7 in patients with complete data and sepsis due to pneumonia

	Day 1	Day 7	p value
Survivors (n=21)			
AGE, ng/mL	4189.1 ± 1466.4	4818.5 ± 1162.9	0.297
sRAGE, pg/mL	2463.2 ± 386.5	1571.7 ± 289.1	0.009
HMGB1, pg/mL	347.1 ± 109.3	339.5 ± 112.5	0.823
S100A12, pg/mL	521.8 ± 27.2	485.5 ± 27.7	0.138
Non-survivors (n=3)			
AGE, ng/mL	1392.0 ± 1352.5	2509.3 ± 2289.5	0.356
sRAGE, pg/mL	1433.6 ± 354.4	908.4 ± 537.0	0.503
HMGB1, pg/mL	99.0 ± 69.1	260.3 ± 157.5	0.209
S100A12, pg/mL	445.8 ± 85.8	587.9 ± 22.4	0.216
All patients (n=24)			
AGE, ng/mL	3839.5 ± 1301.3	4529.8 ± 1054.2	0.200
sRAGE, pg/mL	2334.5 ± 346.5	1488.8 ± 262.4	0.006
HMGB1, pg/mL	316.1 ± 97.2	329.6 ± 99.7	0.687
S100A12, pg/mL	512.3 ± 25.9	498.3 ± 25.3	0.586

Abbreviations: AGE, advanced glycation end products; sRAGE, soluble receptor for AGE; HMGB1, high-mobility group box 1
